# Effect of atorvastatin on lipoxygenase pathway‐related gene expression in an *in vitro* model of lipid accumulation in hepatocytes

**DOI:** 10.1002/2211-5463.13552

**Published:** 2023-03-09

**Authors:** Ivanna Carolina Golfetto Miskiewicz, Hyen Chung Cho, Ji In Lee, Jihye Lee, Yenna Lee, Yun Kyung Lee, Sung Hee Choi

**Affiliations:** ^1^ Translational Medicine Department Seoul National University South Korea; ^2^ Laboratory of Endocrinology and Metabolism, Department of Internal Medicine Seoul National University Bundang Hospital South Korea; ^3^ Department of Internal Medicine Seoul National University College of Medicine South Korea

**Keywords:** ALOX, atorvastatin, cPLA2, hepatosteatosis, lipoxygenase

## Abstract

Lipid accumulation in hepatocytes can result from an imbalance between lipid acquisition and lipid catabolism. In recent years, it has been discovered that eicosanoids derived from arachidonic acid (AA) have the potential to create specialized pro‐resolving lipid mediators to actively resolve inflammation, but it is not clear whether AA and lipoxygenases exert effects on hepatic inflammation. Here, the effects of atorvastatin on the expression of cytoplasmic phospholipase A2 (*cPLA2*) and lipoxygenase pathway genes (*ALOX5*, *ALOX12*, *ALOX15*, and *ALOX15B*) were evaluated in an *in vitro* model of palmitic acid (PA)‐induced hepatocyte lipid accumulation in McA‐RH7777 (McA) cells. Palmitic acid increased *cPLA2* expression, intracellular AA levels, and *ALOX12* expression (*P* < 0.05). Atorvastatin at various concentrations had no significant effects on AA levels or on *cPLA2*, *ALOX15*, and *ALOX15B* expressions. *ALOX5* was not detected, despite multiple measurements. Pro‐inflammatory *IL‐1β* expression levels were upregulated by PA (*P* < 0.01) and attenuated by atorvastatin (*P* < 0.001). *TNFα* did not differ among groups. The expression levels of anti‐inflammatory *IL‐10* decreased in response to PA (*P* < 0.05), but were not affected by atorvastatin. In conclusion, in an *in vitro* model of lipid accumulation in McA cells, atorvastatin reduced *IL‐1β*; however, its effect was not mediated by AA and the lipoxygenase pathway at the established doses and treatment duration. Further research is required to investigate time‐response data, as well as other drugs and integrated cell systems that could influence the lipoxygenase pathway and modulate inflammation in liver diseases.

## Graphical abstract

An *in vitro* hepatic lipid accumulation state in McA‐RH7777 (McA) hepatocytes was achieved by palmitic acid administration, with a particular increase in *cPLA2*, AA and *ALOX12*, as well as a pro‐inflammatory response of *IL‐1β* with a decrease of anti‐inflammatory *IL‐10*. Exposure of McA cells to atorvastatin reduced *IL‐1β*, but did not seem to be mediated by the AA response, while no changes in *cPLA2*, *ALOX12*, *ALOX15*, and *ALOX15B* were observed.

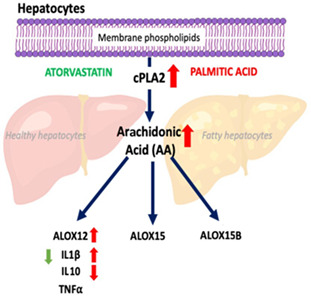

**Abbreviations**
AA: arachidonic acid
BSA: bovine serum albumin
COX: cyclooxygenases
cPLA2: cytosolic phospholipase A2
CYP450: cytochrome P450 enzymes
DMEM: Dulbecco's modified Eagle medium
ELISA: enzyme‐linked immunosorbent assay
FA: fatty acid
FBS: fetal bovine serum
HFD: high‐fat diet
HMGCR: 3‐hydroxy‐3‐methylglutaryl coenzyme A reductase
LOX: lipoxygenase
McA: McARH7777 cells
NAFLD: nonalcoholic fatty liver disease
NASH: nonalcoholic steatohepatitis
PA: palmitic acid
qRT‐PCR: quantitative real‐time polymerase chain reaction
SDS‐PAGE: sodium dodecyl sulfate‐polyacrylamide gel electrophoresis

Lipid accumulation in hepatocytes can result from an imbalance between lipid acquisition and lipid catabolism, causing lipoperoxidative stress and hepatic damage [1]. Although hypercaloric diets (high in fat and carbohydrates) clearly promote liver fat accumulation, the specific effects can differ according to the type of carbohydrate or fat. Fructose and saturated fatty acids induce the highest levels of intrahepatic triglyceride accumulation, insulin resistance, and ceramide production. Thus, avoiding saturated fats and added sugars are important dietary interventions for effectively reducing the risk of type 2 diabetes and associated nonalcoholic fatty liver disease (NAFLD) [2]. Nonalcoholic fatty liver disease is a chronic metabolic disorder involving low‐grade inflammation and liver fat accumulation, with stages that range from hepatocellular steatosis to nonalcoholic steatohepatitis (NASH), fibrosis, and cirrhosis [3]. Palmitic acid (PA), one of the most abundant saturated fatty acids and common in high‐fat diets (HFD), can induce steatosis and inflammation. Therefore, PA is widely used for *in vitro* studies of hepatic steatosis [4].

An interesting link between lipid metabolism and inflammation is arachidonic acid (AA) metabolism, derived from dietary linoleic acid. AA is frequently esterified into the sn‐2 position of phospholipids, conferring membrane fluidity for adequate cell function [5]. AA is de‐esterified and released from the membrane by cytosolic phospholipase A2 (cPLA2), metabolized by lipoxygenases (LOX), cytochrome P450 enzymes (CYP450), and cyclooxygenase (COX), and finally converted into metabolites, such as eicosanoids [6]. Under normal conditions, *ALOX* genes are expressed at low levels in liver tissues [7, 8]. Both human and mouse lipoxygenase (LOX) genes encode 5‐LOX (ALOX5), 12‐LOX (ALOX12), and 15‐LOX (ALOX15), which produce 5‐hydroxyeicosatetraenoic acid (5‐HETE), 12‐HETE, and 15‐HETE from AA [9]. ALOX15B, which synthesizes 15‐HETE, was later identified as another LOX capable of oxygenating AA at carbon 15 [10]. Mouse 15‐LOX predominantly produces 12‐HETE (at a 6 : 1 ratio over 15‐HETE) and is also referred to as 12/15‐LOX [9]. In recent years, researchers have found that eicosanoids derived from AA (n‐6 series) and EPA/DHA (n‐3 series) have the potential to create specialized pro‐resolving lipid mediators, such as lipoxins, to actively resolve inflammation [11]. Nevertheless, the ALOX12 derivative 12‐HETE exhibits pro‐inflammatory activity and may be related to the pathogenesis of NAFLD [9]. 12‐HETE can easily permeate cell membranes, promoting intracellular oxidative stress and interacting with G protein‐coupled receptor 31 (GPR31), thereby modulating inflammation by activating the PI3K/Akt/NF‐κB pathway and increasing TNFα and IL‐1β production [12].

Nonalcoholic fatty liver disease involves multiple cellular actors (Kupffer cells, adipocytes, liver sinusoidal endothelial cells etc.) and complex cell interactions; thus, most studies of NAFLD have been carried out *in vivo*, using human and animal models with or without genetic alterations [13, 14]. Although hepatocytes are the major cell type in the liver [15], few *in vitro* studies of cultured hepatocytes under lipid accumulation have evaluated the expression of lipoxygenase pathway‐related genes and cytokines genes.

Furthermore, effective treatments for NAFLD are still lacking. Statins are among the most prescribed drugs worldwide; they are commonly used for the treatment of hypercholesterolemia, and function by the competitive inhibition of 3‐hydroxy‐3‐methylglutaryl coenzyme A reductase (HMGCR), a rate‐limiting enzyme in cholesterol synthesis [16]. However, statins also exert protective anti‐inflammatory effects on the cardiovascular system [17] and liver [18]. Researchers have examined the pleiotropic effects of statins on the mevalonate and lipogenesis pathways; however, little is known about the effect of statins on the lipoxygenase pathway and related inflammatory and anti‐inflammatory responses. Thus, to address the lack of knowledge about the effect of statins on the lipoxygenase pathway in cultured hepatocytes, the objective of this study was to explore the effect of atorvastatin on lipoxygenase gene expression in a model of lipid accumulation in McA‐RH7777 (McA) cells.

## Methods

### Induction of lipid accumulation in McA cells by palmitic acid

PA (P5585; Sigma, St. Louis, MO, USA) was conjugated to fatty acid‐free bovine serum albumin (BSA) (A0281; Sigma). PA (0.1282 g) was dissolved in 100% ethanol (1 mL) to make a 500 mm stock solution. In Dulbecco's modified Eagle's medium (DMEM), 10% fatty acid‐free BSA was prepared, filtered, and incubated at 37 °C. A volume of 10 μL of 500 mm PA was mixed with 990 μL of 10% fatty acid–free BSA, vortexed, and incubated at 55 °C for 15 min to generate the 10 mm PA stock solution.

A separate experiment was conducted in which McA cells were exposed to different concentrations of PA (50, 100, and 200 μm) for 24 and 48 h to find the dose that induced steatosis with minimal cytotoxicity. Using optical microscopy and quantitative real‐time polymerase chain reaction (qRT‐PCR), 200 μm PA was established as an appropriate dose to achieve lipid accumulation in cells. The effects of statin on gene expression were evaluated at 24 h because the severity of lipid accumulation reduced the viability of cells at 48 h.

### Cell culture and statin treatment

McA cells were purchased from the American Type Culture Collection (Manassas, VA, USA). Cells were grown in DMEM (11995065; Sigma) supplemented with 10% fetal bovine serum (FBS) and 2% 100 units·mL^−1^ penicillin–streptomycin (Invitrogen, Carlsbad, CA, USA) at 37 °C, 5% CO_2_, and 95% humidity. McA cells were seeded in six‐well plates at a density of 2 × 10^5^ cells per well with DMEM (containing 10% FBS and 2% penicillin–streptomycin) and incubated at 37 °C, 5% CO_2_, and 95% humidity for 24 h to allow cell attachment. The 10 mm palmitic acid‐BSA conjugate in 10% BSA was added to DMEM without 10% FBS to prepare 200 μm PA.

Atorvastatin dosages for cell culturing were calculated based on human dosages. Thus, human doses of 10, 40, and 80 mg of atorvastatin was calculated and approximated to 10, 30, and 65 μm for cell culture use, respectively.

Using atorvastatin calcium (PHR1422; Sigma), a 20 mm stock solution was prepared in 100% DMSO. The final working solution of atorvastatin (10, 30, and 65 μm) was prepared with DMSO (Sigma‐Aldrich; final concentration of 0.1% in DMEM). Cells were treated with PA for 24 h, washed with PBS to remove PA, and treated with DMEM (11995065; Sigma) supplemented with 10% FBS and 2% 100 units·mL^−1^ penicillin–streptomycin (Invitrogen) and atorvastatin for 24 h.

### RNA isolation and quantitative real‐time PCR

Total RNA was extracted from McA cells using TRIzol (Ambion, Foster City, CA, USA) 1 μg of total RNA was reverse‐transcribed using a cDNA Reverse Transcription Kit (Thermo Fisher Scientific, Waltham, MA, USA). SYBR Green reactions using SYBR Green PCR Master Mix (Enzynomics, Daejeon, Korea) were assembled along with 10 pm primers according to the manufacturer's instructions and were performed using the Applied Biosystems ViiA7 system (Thermo Fisher Scientific). Relative mRNA levels were calculated with the comparative CT method and normalized to GAPDH. Sequences of all primers are listed in Table 1.

**Table 1:** Primers used to assess mRNA transcript abundance in McA cells. RT‐qPCR, quantitative reverse transcription PCR.

Gene symbol		Primer sequence	
Rat genes	Accession no.	Forward primer (5′‐3′)	Reverse primer (5′‐3′)
GAPDH	–	GACTCTACCCACGGCAAGTT	GGTGATGGGTTTCCCGTTGA
cPLA2	>NM_133551.2	CTGGGGCAGTGCCTTTTCTA	CGCTGTCAGAGCTGTCGTTA
ALOX5	>NM_012822.2	TGTACACACCAGTTCCTGGC	TGGCCAAAAGCCAGTCGTAT
	>NM_012822.2	CCACCGGTAGCCAGTGGTTC	CCGCGCTCGAAGTCATTGTA
ALOX12	–	GGCTATCCAGATTCAGCCTCC	ATGGTGGCAACAGCAATGAC
ALOX15	>NM_031010.2	GAGACTCCAAGTACGCGGGC	GAATTCTGCTTCCGAGTCCCG
ALOX15B	>NM_153301.2	GTGCAAGCCAGTTTGACTCG	CATGTGGCATTGACTGCTGG
TNFα	–	TGGGCTTTCGGAACTCACTG	CTGTGCCTCAGGGAACAGTC
IL‐1β	–	GACTTCACCATGGAACCCGT	GGAGACTGCCCATTCTCGAC
IL‐10	>NM_012854.2	CATTCCATCCGGGGTGACAA	TGTTGTCCAGCTGGTCCTTC

### Determination of AA levels

McA cells treated with PA for 24 h and then atorvastatin at 10, 30, and 65 μm were harvested and homogenized. The supernatant was collected and measured by an enzyme‐linked immunosorbent assay (ELISA) using the Rat Arachidonic Acid (AA) ELISA Kit (Cusabio, Houston, TX, USA), according to the manufacturer's instructions.

### Triglyceride quantification

McA cells treated with PA and then atorvastatin, as described above, were scraped from wells using 5% TritonX‐100 and transferred to Eppendorf tubes for homogenization. From each sample, 50 μL was aliquoted for further protein estimation assays. Samples were then placed on a heating block at 30 °C increased to 80 °C for 30 min, cooled on ice for 10 min, and vortexed, and then the process was repeated. Debris were pelleted using a Beckman Coulter Microfuge 20R centrifuge (13201 × ***g***, 5 min, 20 °C). Of standards solution and sample supernatants, 50 μL was transferred to a 96‐well microtiter plate. Prewarmed triglyceride solution (981786; Thermo Fisher Scientific) was added to each sample and standard and then incubated for 30 min at 37 °C in a water bath. Total absorbance was measured at 540 nm and corrected by subtraction of the value for the blank. Substrate absorbance was evaluated prior to triglyceride equivalent content calculation using 0–40 μg of triolein (T7140; Sigma) as the TAG standard.

### Western blot analysis

Proteins were extracted from cells, and the lysates were resolved by 10% SDS/PAGE (sodium dodecyl sulfate‐polyacrylamide gel electrophoresis), followed by transfer to nitrocellulose membranes. Membranes were incubated with primary antibodies for cPLA2 (SC‐454; Santa Cruz Biotechnology, Dallas, TX, USA) and β‐actin (A5441; Sigma‐Aldrich) overnight at 4 °C. Then, the membranes were washed and incubated with horseradish peroxide‐conjugated goat anti‐mouse IgG secondary antibodies (SC‐2031; Santa Cruz Biotechnology) for 1 h at room temperature. The densitometric quantification of the protein bands was performed using imagej version 1.53 e (National Institutes of Health, Bethesda, MD, USA).

### Statistical analysis

Statistical analyses were performed using graphpad prism (version 8.0; GraphPad Software, La Jolla, CA, USA). Data for McA cells were compared using one‐way analysis of variance (ANOVA). Triglyceride levels were assessed using nonparametric Wilcoxon signed‐rank test to evaluate differences in lipid accumulation among samples. Values of *P* < 0.05 were regarded as statistically significant.

## Results

### Effects of palmitic acid and atorvastatin on intracellular lipid accumulation in McA cells

**Fig. 1:**
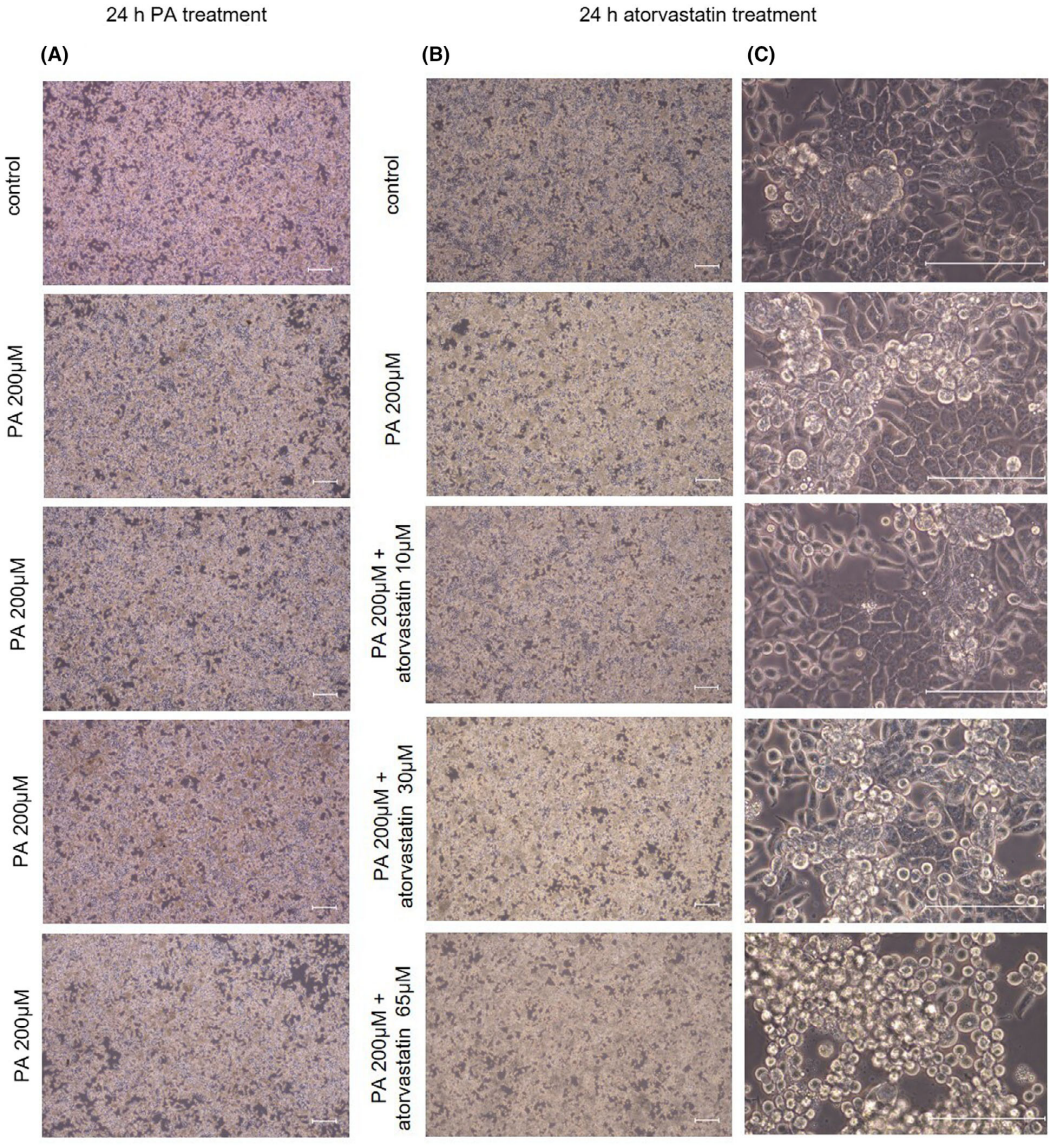
Image analysis of lipid accumulation in McA cells after exposure to 200 μm PA for 24 h, followed by treatment with 10, 30, or 65 μm atorvastatin for 24 h. (A, B) Light microscopy images. Scale bar = 100 μm. (C) Light microscopy images. Scale bar = 50 μm. PA, palmitic acid.

**Fig. 2:**
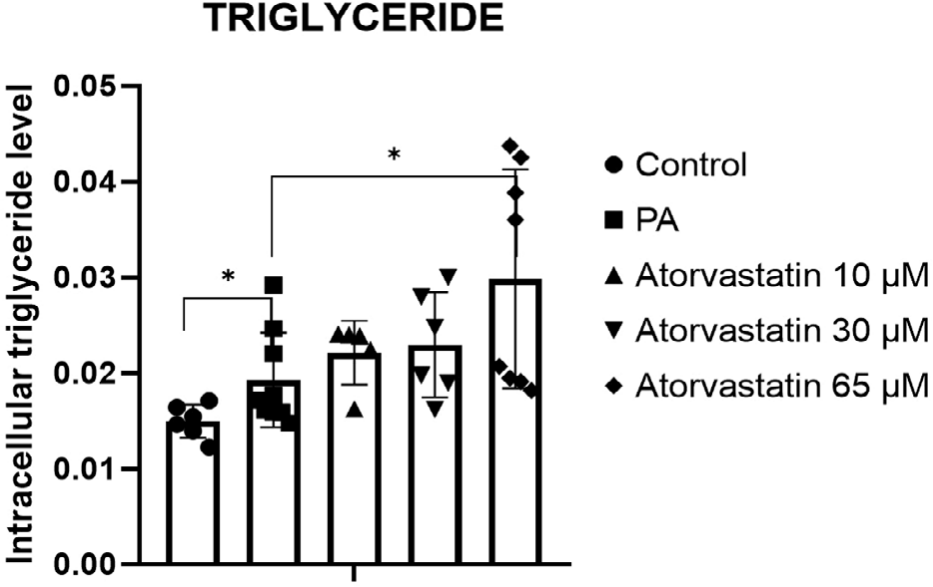
**I**ntracellular triglyceride levels in McA cells after treatment with 200 μm PA for 24 h, followed by treatment with 10, 30, and 65 μm atorvastatin for 24 h. Significant increase in triglyceride levels in response to PA and atorvastatin at 65 μm. Triglyceride was normalized to protein concentration. Results are expressed as mean ± SD (*n* = 9) from three independent experiments. *P < 0.05 calculated by Wilcoxon signed‐rank test. PA, palmitic acid.

In our previous experiments, lipid accumulation and gene expression of *cPLA2*, *ALOX12*, *ALOX15*, and *ALOX15B* did not differ between McA cells treated with DMSO at a final concentration of 0.1% for 24 h and the control group (data not shown). Levels of intracellular lipid accumulation in McA cells treated with 200 μm PA for 24 h (Fig. 1A) and with atorvastatin at 10, 30, or 65 μm for 24 h (Fig. 1B,C) were visualized under a light microscope with magnifications of 40× and 400×. Atorvastatin led to dose‐dependent increases in lipid accumulation, with a substantial increase at 65 μm. These findings based on visual assessment were confirmed by the quantification of triglyceride levels. The levels of triglyceride increased by PA and by atorvastatin in a dose‐dependent manner. Triglyceride levels were significantly increased with PA in comparison with the control group (*P* < 0.05), while 10 and 30 μm atorvastatin showed a tendency to increase compared with levels in the PA group. The greatest increase was observed in the group treated with 65 μm atorvastatin (*P* < 0.05; Fig. 2).

### Effects of palmitic acid and atorvastatin on gene and protein expression levels in McA cells

**Fig. 3:**
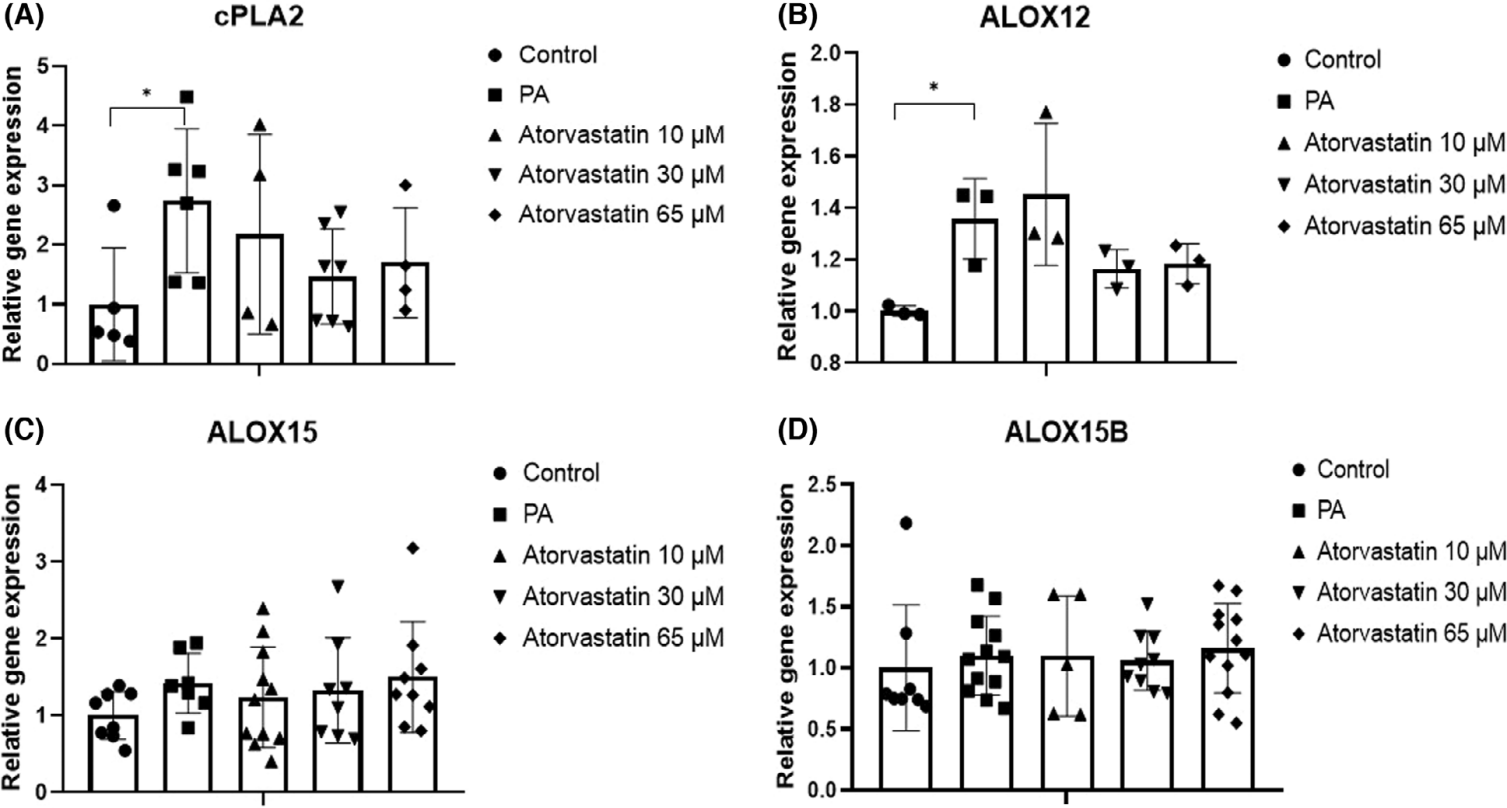
Effects of 10, 30, and 65 μm atorvastatin on levels of *cPLA2* and lipoxygenase pathway‐related genes in McA cells. (A) PA significantly increased *cPLA2* expression. (B) PA significantly increased *ALOX12* expression. (C, D) Treatment with PA alone or atorvastatin did not affect *ALOX15* and *ALOX15B* expression. Relative expression levels of target genes are expressed as mean ± SD (*n* = 3–5) from four independent experiments. *P < 0.05, calculated by one‐way ANOVA. *ALOX12*, Arachidonate 12‐Lipoxygenase; *ALOX15*, Arachidonate 15‐Lipoxygenase; *ALOX15B*, Arachidonate 15‐lipoxygenase type B; *cPLA2*, Cytosolic phospholipase A2.

The role of cPLA2 in cleaving AA from phospholipids prompted us to evaluate mRNA and protein expression levels of this gene. *cPLA2* mRNA expression levels in McA cells were higher in the PA group compared to the control group (*P* < 0.05); however, treatment with atorvastatin at various doses did not result in significant *cPLA2* expression changes when compared to PA (Fig. 3A). Protein expression levels of cPLA2 did not differ among groups, although an analysis of band intensities demonstrated an increase in cPLA2 expression in response to PA and slight decreases in response to atorvastatin compared with levels in the PA group (Fig. 6A,B).

*ALOX12* encodes the 12‐LOX enzyme, which catalyzes the conversion of AA into its product, 12‐HETE, an inflammatory signaling molecule in the lipoxygenase pathway. When cultured hepatocytes were incubated with PA, there was an increase in the expression of *ALOX12* (*P* < 0.05). When cells were exposed to atorvastatin at all doses, there was no evident decrease in *ALOX12* gene expression (Fig. 3B).

*ALOX15* and *ALOX15B* encode 15‐LOX and 15‐LOX‐2, which produce 15‐HETE and 15(S)‐HPETE, respectively, mediators involved in inflammation derived from AA. These genes did not show alterations in relative expression when exposed to PA and statin (Fig. 3C,D). Despite repeated measurements, *ALOX5*, which encodes an AA derived product (5‐HETE), was not detected in McA cells.

**Fig. 4:**
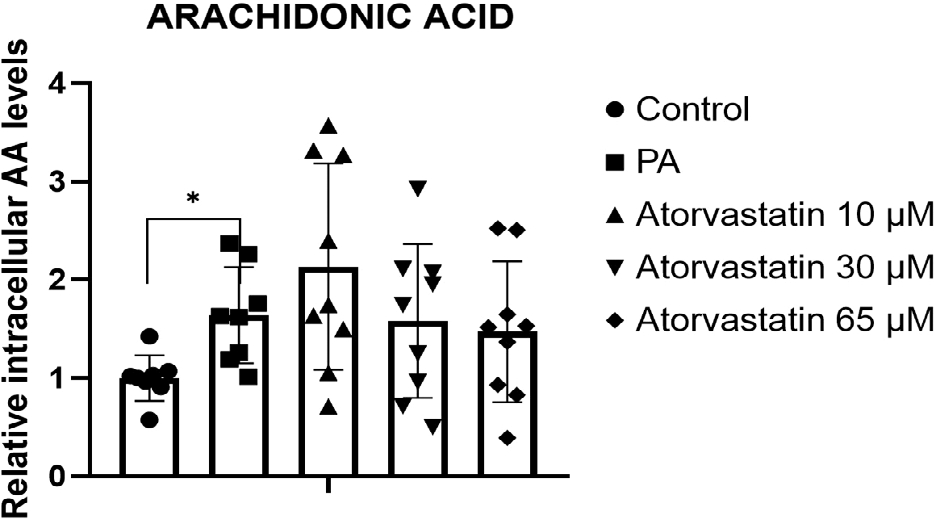
Effects of 10, 30, and 65 μm atorvastatin on relative intracellular levels of arachidonic acid (AA) in McA cells. PA significantly increased AA levels. AA was normalized to protein concentration. Results are expressed as mean ± SD (*n* = 9) from three independent experiments. *P < 0.05 calculated by one‐way ANOVA. AA: arachidonic acid; PA, palmitic acid.

To better understand the link between cPLA2 and ALOX, intracelullar levels of AA were measured by ELISA. The intracelullar levels of AA showed a significant increase in response to PA (*P* < 0.05); however, AA levels did not differ between groups treated with atorvastatin in comparison with levels in the PA group (Fig. 4).

**Fig. 5:**
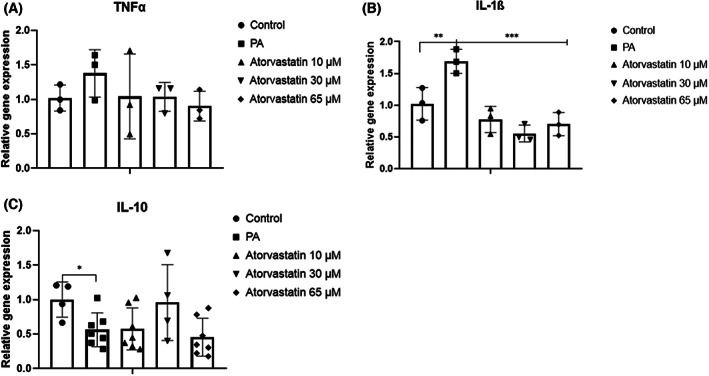
Effects of 10, 30, and 65 μm atorvastatin on the expression pro‐inflammatory and anti‐inflammatory response‐related genes in McA cells. (A) No statistically significant changes in *TNFα* expression were detected. (B) Increase *IL‐1β* expression levels were increased by PA and decreased by atorvastatin at all doses. (C) PA significantly decreased *IL‐10* expression. Tendency to increase *IL‐10* expression with atorvastatin at 30 μm (*P* = 0.06). Relative expression levels of target genes are expressed as mean ± SD (*n* = 3–5) from four independent experiments. *P < 0.05, **P < 0.01, ***P < 0.001 calculated by one‐way ANOVA. *IL‐10*, interleukin‐10; *IL‐1β*, interleukin‐1β; PA, palmitic acid; *TNFα*, tumor necrosis factor alpha.

To confirm whether PA and statins affect inflammation and the anti‐inflammatory response, *TNFα*, *IL‐1β*, and *IL‐10* gene expression levels were evaluated. In the presence of PA, pro‐inflammatory *TNFα* expression levels showed a tendency to increase but did not differ significantly from expression levels in the control group; however, *TNFα* expression levels tended to decrease in response to atorvastatin at all doses (Fig. 5A). *IL‐1β* gene expression levels were significantly higher in the PA group than in the control group (*P* < 0.01) and were lower in the groups treated with atorvastatin at all doses than in the PA group (*P* < 0.001) (Fig. 5B). As a conterpart, expression levels of the anti‐inflammatory gene *IL‐10* were lower in the PA group than in the control group (*P* < 0.05) and tended to be higher in the group treated with atorvastatin at 30 μm than in the PA group (*P* = 0.06; Fig. 5C).

**Fig. 6:**
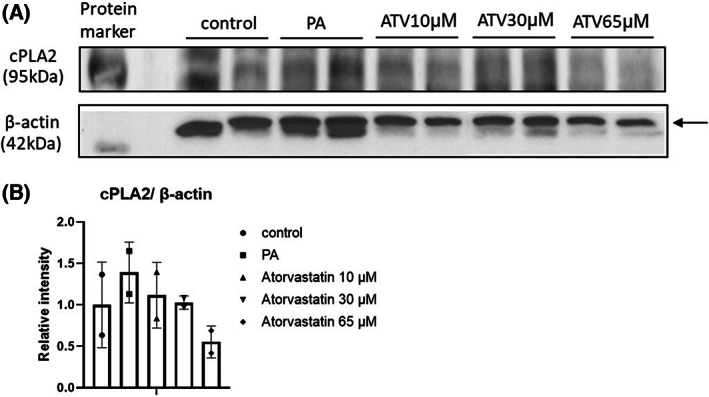
Effects of atorvastatin on intracellular levels of cPLA2 in McA cells. (A) cPLA2 expression decreased in response to atorvastatin at 10, 30, and 65 μm, as determined by an immunoblot analysis. (B) Graphical representation of the increased relative intensity after treatment with PA and dose‐dependent decrease after treatment with atorvastatin using imagej (NIH). β‐Actin was used as a control for band normalization. Results are expressed as mean ± SD. ATV, atorvastatin; Beta Actin, β‐Actin; cPLA2, Cytosolic phospholipase A2; PA, palmitic acid.

Therefore, we presume that under lipid accumulation, cPLA2 increment might be related to increasing levels of AA, which is required for the synthesis of 12‐HETE by *ALOX12*, and may also be related to the increase in *IL‐1β* gene expression. However, although atorvastatin reduced *IL‐1β* gene expression at all doses, its effects were not mediated by *ALOX12*, suggesting that the pleiotropic functions of atorvastatin are mediated by a different mechanism.

## Discussion

Statins have anti‐inflammatory effects, especially in cardiovascular tissues, where the activation of pro‐resolving mediators plays a crucial role [19]. However, few studies have explored whether statins can induce ALOX‐derived mediators to reduce the inflammatory response in the liver and thereby to resolve chronic metabolic diseases, such as NAFLD [20].

The first step in this study was to establish an *in vitro* model of hepatocyte lipid accumulation using PA as the main source of FA. As determined by histological observations, control McA cells demonstrated typical cell characteristics, while those cells exposed to PA showed increased storage of intracellular lipids (a hallmark of hepatic steatosis). After statin exposure, dose‐dependent lipid accumulation was observed, with the highest accumulation at the highest dose, 65 μm atorvastatin (Fig. 1). In a quantitative analysis, triglyceride levels were significantly increased after treatment with PA *vs* the control group, suggesting that PA resulted in a substantial accumulation of intracellular lipids. Atorvastatin resulted in a dose‐dependent increase in triglyceride levels, with a significant increase at 65 μm *vs* PA group. It is possible that the inhibition of HMG‐CoAR results in the accumulation of acetyl‐CoA and acetoacetyl‐CoA, which serve as building components for triglyceride synthesis. Similar results have been obtained using simvastatin by Gbelcová et al., in which HEK 293T cells incubated with and without FBS exhibited a marked increase in cytosolic lipid droplets and significant increases in the expression of two genes involved in the *de novo* synthesis of phospholipids and triglycerides [21]. In our study, considering the pleiotropic effects of statins and the significant reduction of *IL‐1β* observed in response to atorvastatin, we assume that lipid accumulation in McA cells could be consistent with a cytoprotective effect. Recent studies have suggested that when FA accumulates in RINm5F and INS‐1 E cells, it is channeled into cytosolic lipid droplets as a protective mechanism to reduce lipotoxicity and insulin resistance [15, 22, 23, 24]. Similar conclusions have also been obtained for other drugs, such as dexamethasone evaluated in primary hepatocytes treated with PA. Contrary to other types of cells, such as skeletal muscle cells, hepatocytes may induce the redistribution of FA toward triglyceride synthesis, in addition to intensifying the export of excessive lipids [25].

We further evaluated the importance of membrane lipids and the effect of PA on cells. To maintain plasmatic membrane fluidity, FA phospholipids, particularly AA, is required [26]. *cPLA2* catalyzes the hydrolysis of AA from phospholipids, which is the precursor of bioactive lipids in the LOX pathway. In our experimental model using PA, we detected significant increases in *cPLA2* expression at the gene and protein levels as well as, an increase in AA levels. Previous studies have described a phenomenon known as membrane remodeling, in which 60–70% of PA influx is predominantly incorporated into the *sn*‐2 position of phosphatidylcholine and phosphatidylethanolamine [27], removing AA from the membrane and replacing it with PA [28]. As demonstrated by Bolognesi et al., in neuroblastoma cells, this can lead to cell signaling alterations, caspase activation, and increased *cPLA2* expression only 15 min after the administration of 150 μm PA. Within 1 h, a noticeable decrease in the membrane AA composition was observed. Thus, in this previous study, increased PA influx led to a rapid increase in AA release from the membrane by *cPLA2* [29].

Additionally, we studied the expression of genes related to the lipoxygenase pathway. Under normal conditions, *ALOX* genes show low expression levels in liver tissues [7, 8]. In our study, levels of *ALOX15* and *ALOX15B* did not differ among groups (i.e., they were not influenced by PA nor statins). The role of *ALOX15* in mice is controversial, with evidence for both pro‐ and anti‐inflammatory properties. A study of whole‐body *Alox15* ^*−/−*^ mice under a HFD revealed a decrease in lymphocyte infiltration and pro‐inflammatory mRNA levels of *TNFα* and *IFN‐γ*, thereby protecting against hepatic steatosis [30]. We did not detect *ALOX5*, suggesting that it is expressed at very low levels in McA cells. Ideally, this result should be confirmed by metabolite determination in future studies. *ALOX12* expression was significantly upregulated in the presence of PA. The ALOX12‐derived metabolite 12‐HETE modulates the activation of PI3K/Akt/NF‐κB and increases pro‐inflammatory *TNFα* and *IL‐1β* production [12] in liver ischemia–reperfusion injury and in NAFLD [31].

Thus, we also evaluated pro‐ and anti‐inflammatory mediators. PA tended to increase pro‐inflammatory *TNFα* expression levels and significantly increased *IL‐1β* expression levels. *IL‐1β* constitutes one of the most important cytokines in the liver and plays a critical role in inflammation via autocrine and paracrine activity; it is markedly elevated in chronic liver disease [32]. In primary hepatocytes, *IL‐1β* is associated with triglyceride and cholesterol accumulation and the development of hepatic steatosis and lipid droplet formation [33]. Simultaneously, we found a decrease in anti‐inflammatory *IL‐10* expression. *IL‐10* has been associated with the anti‐inflammatory response and the amelioration of hepatocellular damage. It has been suggested that *IL‐10* expression levels are decreased in nonalcoholic steatohepatitis (NASH) and the worsening of pro‐inflammatory markers and insulin resistance when neutralizing anti‐IL‐10 antibodies have been used in a diet‐induced mouse model of NAFLD [34]. Collectively, these results suggest that under lipid accumulation, a connection between increased *cPLA2* expression and increased AA intracellular levels may lead to increased *ALOX12* and *IL‐1β* expression.

To evaluate the effect of statins, after PA exposure, atorvastatin was administered at three concentrations (10, 30, and 65 μm, approximately equivalent to human doses of 10 mg, 40 mg, and 80 mg, respectively). Atorvastatin tended to decrease AA intracellular levels and *cPLA2* and *ALOX12* gene expression levels; however, the differences among groups were not significant. Western blotting also revealed a decrease in *cPLA2* band intensity with increasing concentrations of atorvastatin, with no significant changes in AA levels.

Likewise, no significant changes in *ALOX15* and *ALOX15B* gene expression were observed in response to atorvastatin. Thus, the synthesis of lipoxygenases derived from AA did not contribute to the anti‐inflammatory effects of atorvastatin. Further studies accounting for time‐dependent responses to statins and interactions with nonparenchymal cells involved in fatty liver development, including NAFLD, are warranted.

Although no modifications in *ALOX* genes were observed, the administration of atorvastatin tended to reduce inflammatory gene expression levels. Specifically, *IL‐1β* expression levels decreased significantly, whereas anti‐inflammatory *IL‐10* levels tended to increase in response to atorvastatin at 30 μm (*P* = 0.06). Although hepatocytes are not inflammatory cells, they express certain cytokines that are autoregulatory or influence the functions of other liver cells, such as Kupffer or endothelial cells [35]. HepG2 cells express *TNFα* and *IL‐10* [35]. Studies of murine primary hepatocytes exposed to PA have also described a moderate increase in *IL‐1β* mRNA expression [36]. Even though statins have been found to decrease hepatic inflammation in whole tissues by decreasing pro‐inflammatory cytokines, few studies have clarified the effect of statins on inflammation in a single type of liver cell. A histological analysis of hepatic tissue in rats fed a HFD demonstrated that rosuvastatin decreases hepatic inflammation by inhibiting the expression of *TNFα* and *IL‐1β* [37]. Although it has been suggested that *IL‐10* in the liver can inhibit *TNFα* production and protect against hepatic steatosis during HFD, no improvement in insulin sensitivity has been found [38]. The effects of statins are regulated, in part, by pathways mediated by Rho/ROCK via the inhibition of isoprenoid intermediates from the mevalonate pathway [39]. Hence, in our study, although atorvastatin decreased *IL‐1β* expression levels, the lack of changes in *cPLA2* and *ALOX12* expression and in AA levels suggested that the effects are mediated by an alternative intracellular pathway.

## Conclusions

In conclusion, in an *in vitro* model of PA‐induced lipid accumulation, we demonstrate an inflammatory process involving significant increases in the expression of *ALOX12*, *cPLA2*, and *IL‐1β* and levels of AA and a simultaneous decrease in IL‐10 levels. Other than the reduction in inflammatory *IL‐1β*, atorvastatin did not seem to contribute to decreases in the pro‐inflammatory response via the lipoxygenase pathway. Further research is needed to explore time‐response data, other drugs, and integrated cell systems representative of NAFLD and the effects on the lipoxygenase pathway for the regulation of inflammation in liver diseases.

## Conflict of interest

The authors declare no conflict of interest.

## Author contributions

IGM, YKL, and SHC were involved in conceptualization; IGM, YKL, and SHC were involved in methodology; IGM was responsible for software; IGM, YKL, and SHC were involved in validation; IGM was involved in formal analysis; IGM, HC, JL, and JIL were involved in investigation; YKL and SHC were involved in resources; IGM was involved in data curation; IGM was involved in writing—original draft preparation; IGM, YL, YKL, and SHC were involved in writing—review and editing; IGM, YKL, and SHC were involved in visualization; YKL and SHC were involved in supervision; YKL and SHC were involved in project administration; YKL and SHC were involved in funding acquisition. All authors have read and agreed to the published version of the manuscript.

## Data Availability

The data that support the findings of this study are available from the corresponding author, [YKL and SHC], upon reasonable request.
